# Interferon signaling and hypercytokinemia-related gene expression in the blood of antidepressant non-responders

**DOI:** 10.1016/j.heliyon.2023.e13059

**Published:** 2023-01-16

**Authors:** Hirotaka Yamagata, Ryouichi Tsunedomi, Toshiharu Kamishikiryo, Ayumi Kobayashi, Tomoe Seki, Masaaki Kobayashi, Kosuke Hagiwara, Norihiro Yamada, Chong Chen, Shusaku Uchida, Hiroyuki Ogihara, Yoshihiko Hamamoto, Go Okada, Manabu Fuchikami, Jun-ichi Iga, Shusuke Numata, Makoto Kinoshita, Takahiro A. Kato, Ryota Hashimoto, Hiroaki Nagano, Shuichi Ueno, Yasumasa Okamoto, Tetsuro Ohmori, Shin Nakagawa

**Affiliations:** a Division of Neuropsychiatry, Department of Neuroscience, Yamaguchi University Graduate School of Medicine, 1-1-1 Minami-Kogushii, Ube, Yamaguchi 755-8505, Japan; b Kokoro Hospital Machida, 2140 Kamioyamadamachi, Machida, Tokyo 194-0201, Japan; c Department of Gastroenterological, Breast and Endocrine Surgery, Yamaguchi University Graduate School of Medicine, 1-1-1 Minami-Kogushii, Ube, Yamaguchi 755-8505, Japan; d Department of Psychiatry and Neurosciences, Graduate School of Biomedical and Health Sciences, Hiroshima University, 1-2-3 Kasumi, Minami-ku, Hiroshima 734-8551, Japan; e SK Project, Medical Innovation Center, Kyoto University Graduate School of Medicine, 53 Shogoin Kawahara-cho, Sakyo-ku, Kyoto 606-8507, Japan; f Division of Electrical, Electronic and Information Engineering, Graduate School of Sciences and Technology for Innovation, Yamaguchi University, 2-16-1 Tokiwadai, Ube, Yamaguchi 755-8611, Japan; g Department of Computer Science and Electronic Engineering, National Institute of Technology, Tokuyama Collage, Gakuendai, Shunan, Yamaguchi, Japan; h Department of Neuropsychiatry, Molecules and Function, Ehime University Graduate School of Medicine, Shitsukawa, Toon, Ehime 791-0295, Japan; i Department of Psychiatry, Graduate School of Biomedical Sciences, Tokushima University, 3-18-5 Kuramoto-cho, Tokushima 770-8503, Japan; j Department of Neuropsychiatry, Graduate School of Medical Sciences, Kyushu University, 3-1-1 Maidashi, Higashi-ku, Fukuoka 812-8582, Japan; k Department of Pathology of Mental Diseases, National Institute of Mental Health, National Center of Neurology and Psychiatry, 4-1-1 Ogawa-Higashi, Kodaira, Tokyo 187-8553, Japan

**Keywords:** Antidepressant, Biomarkers, Gene expression, Hypercytokinemia, Interferon, Peripheral blood

## Abstract

Only 50% of patients with depression respond to the first antidepressant drug administered. Thus, biomarkers for prediction of antidepressant responses are needed, as predicting which patients will not respond to antidepressants can optimize selection of alternative therapies. We aimed to identify biomarkers that could predict antidepressant responsiveness using a novel data-driven approach based on statistical pattern recognition. We retrospectively divided patients with major depressive disorder into antidepressant responder and non-responder groups. Comprehensive gene expression analysis was performed using peripheral blood without narrowing the genes. We designed a classifier according to our own discrete Bayes decision rule that can handle categorical data. Nineteen genes showed differential expression in the antidepressant non-responder group (n = 15) compared to the antidepressant responder group (n = 15). In the training sample of 30 individuals, eight candidate genes had significantly altered expression according to quantitative real-time polymerase chain reaction. The expression of these genes was examined in an independent test sample of antidepressant responders (n = 22) and non-responders (n = 12). Using the discrete Bayes classifier with the *HERC5, IFI6,* and *IFI44* genes identified in the training set yielded 85% discrimination accuracy for antidepressant responsiveness in the 34 test samples. Pathway analysis of the RNA sequencing data for antidepressant responsiveness identified that hypercytokinemia- and interferon-related genes were increased in non-responders. Disease and biofunction analysis identified changes in genes related to inflammatory and infectious diseases, including coronavirus disease. These results strongly suggest an association between antidepressant responsiveness and inflammation, which may be useful for future treatment strategies for depression.

## 1 Introduction

Depression is associated with repeated relapses and remissions. The 12-month prevalence of major depressive disorder (MDD) is approximately 6%, and the lifetime risk of depression is 15–18% [1]. In one-third of patients, depression is chronic and refractory to treatment, resulting in a significant reduction in quality of life [1,2]. Selective serotonin-reuptake inhibitors (SSRIs), which are widely used as first-line antidepressants, have a response rate of 39.6–68.0% [2] and remission rate of 23.5% [3]. The STAR*D study, a large-scale practical clinical trial, reported a response rate of 48.6% and remission rate of 36.8% in patients with depression who received an antidepressant as their first treatment step [4]. Given the low response rates, biological indicators that can predict the antidepressant response are desirable. If patients with depression who will not respond to antidepressants could be identified in advance, alternative therapies such as electroconvulsive therapy, repetitive transcranial magnetic stimulation, or cognitive behavioral therapy could be implemented.

A recent large-scale genome-wide association study (GWAS) identified three single-nucleotide polymorphisms related to antidepressant responsiveness, and a gene-enrichment analysis identified immune response genes [5]. However, other GWASs have reported that no antidepressant treatment response-related genes meeting genome-wide significance could be identified; thus, no consensus was established [6,7].

The expression of proinflammatory genes, including interleukin 1 beta (*IL1B*) and tumor necrosis factor alpha (*TNFA*), is related to treatment response [8]. A recent review describing antidepressant response candidate gene expression studies in peripheral blood revealed that mRNA levels of *IL1B*, *IL11*, *TNFA*, and FK506-binding protein 5 (*FKBP5*) may be potential predictive and mediator biomarkers [9]. In a comprehensive analysis without a prior hypothesis, the expression of genes related to inflammatory responses was increased in citalopram non-responders [10]. In another report, changes in the expression of genes involved in the immune response and inflammation were associated with antidepressant responses [11]. A study of plasma proteins related to treatment prognosis for depression indicated that cytokines, such as interleukins, are involved in treatment responsiveness [12]. However, the results of individual gene expression levels are not consistent among studies [[13], [14], [15], [16]]. Therefore, further data accumulation is needed. In addition, to the best of our knowledge, only one study has reported the results of a comprehensive gene analysis validated by another method, such as quantitative real-time polymerase chain reaction (q-PCR), and in independent samples with further pathway analysis [11].

Thus, the present study aimed to comprehensively analyze gene expression in peripheral blood and identify biomarkers that could predict antidepressant treatment responsiveness using a novel data-driven approach based on statistical pattern recognition. We also aimed to examine the pathophysiology of the treatment response via pathway analysis of the identified genes.

## 2 Methods

### 2.1 Ethical approval

This study was conducted in accordance with the principles embodied in the latest version of the Declaration of Helsinki. The Ethics Committee of Hiroshima University (approval number H-35), the Institutional Ethics Committee of the University of Tokushima Graduate School (approval number R3-24), and the Institutional Review Board of the Yamaguchi University Hospital (approval number H30-172) approved this study. All participants provided informed consent before participation.

### 2.2 Participants

Participant set 1: Thirty participants were recruited from the Hiroshima University Hospital and collaborating clinics between 2012 and 2018 for the Depression Biomarker Project approved by the Ethics Committee of Hiroshima University. Patients with MDD were diagnosed by trained psychiatrists following the Diagnostic and Statistical Manual of Mental Disorders, Fourth Edition (DSM-IV) criteria using unstructured interviews, information from medical records, and use of the Mini-International Neuropsychiatric Interview (M.I.N.I.) [17] by a research psychiatrist. All participants could provide written informed consent because the severity of depressive symptoms was moderate. No patient had taken any antidepressants for ≥1 month preceding their inclusion in the study.

Participant set 2: Sixteen unmedicated patients with MDD were recruited from Tokushima University Hospital between 2004 and 2012 for the Research on Analysis of Genes for Mental Disorders study, which has been approved by the Institutional Ethics Committee of the University of Tokushima Graduate School. MDD was diagnosed based on the DSM-IV criteria by at least two trained psychiatrists. None of the patients had any other psychiatric disorders (axis I or II). The exclusion criteria were the use of non-steroidal anti-inflammatory agents, steroids, antidepressants, or anticonvulsants at the time of blood collection.

Participant set 3: Eighteen patients with MDD were recruited at Yamaguchi University Hospital between 2019 and 2020 using community posters for the Depression Stratification Project. This project was approved by the Institutional Review Board of Yamaguchi University Hospital. The diagnosis of MDD was based on the DSM-5 criteria by trained psychiatrists. Participants were screened using the Japanese version of the M.I.N.I. and/or by clinical interview. The exclusion criteria included current or previous substance abuse/dependence, other psychotic illnesses, any neurological disease, family history of hereditary neurological disorder, endocrine disease, head trauma, or other severe medical conditions (e.g., liver failure).

The depressive symptoms of all participants were assessed using the Structured Interview Guide for the Hamilton Depression Rating Scale (SIGH-D) [18,19] before and after treatment in accordance with the planned evaluation period (set 1: 6–8 weeks, set 2: 8 weeks, set 3: 4–12 weeks). Participants whose scores improved by ≥ 50% compared with their pre-treatment scores were defined as the responder group, and those whose scores improved by <50% were defined as the non-responder group. The demographic information of all participants is shown in Table 1. The main classes of antidepressants used are shown in Supplementary Table 1.

**Table 1:** Demographic characteristics of participants.

	Group	Number	Sex (Male/Female)	Age (years)	SIGH-D-pre	SIGH-D-post	Equivalent dose of IMI-pre (mg)	Equivalent dose of IMI-post (mg)
Participant set 1	RES	15	8/7	36.5 ± 7.3	19.3 ± 3.3	5.8 ± 2.8*	0	83.3 ± 47.4
	NRES	15	6/9	37.3 ± 8.7	20.7 ± 5.6	15.4 ± 4.0	0	110.0 ± 50.0
Participant set 2	RES	11	3/8	43.3 ± 15.8	23.8 ± 6.3	5.7 ± 4.2*	0	100.2 ± 53.8
	NRES	5	0/5	40.6 ± 17.0	25.4 ± 4.2	18.6 ± 5.7	0	127.5 ± 33.5
Participant set 3	RES	11	8/3	56.0 ± 19.3	21.3 ± 6.9	4.4 ± 3.0*	80.7 ± 132.7	106.8 ± 79.7
	NRES	7	2/5	65.0 ± 12.7	19.4 ± 9.4	17.6 ± 5.9	117.9 ± 92.9	196.4 ± 89.2

Data are shown as mean ± standard deviation.

*P < 0.05 (unpaired Student’s t-test).

Abbreviations: RES, responder; NRES, non-responder; SIGH-D, Structured Interview Guide for the Hamilton Depression Rating Scale; IMI, imipramine.

### 2.3 RNA isolation from human blood samples

Participant set 1: Total RNA was isolated from blood samples following our previous study [20]. In brief, for each participant, 7 mL of venous blood sample was collected in EDTA-Na_2_ tubes and then stored in a deep freezer at −80 °C for several years. Once thawed, the blood samples were immediately mixed with the lysis buffer of the NucleoSpin RNA blood kit (Takara Bio, Kusatsu, Japan). RNA was isolated following the protocol established by the manufacturer.

Participant sets 2 and 3: At the Tokushima University Hospital and Yamaguchi University Hospital, venous blood samples were collected from participants into PAXgene tubes (Becton, Dickinson and Company, Franklin Lakes, NJ, USA). RNA isolation from the filled PAXgene tubes was performed using the PAXgene Blood RNA Kit (Qiagen, Venlo, Netherlands) following the protocol established by the manufacturer.

The quality and quantity of RNA were measured with a NanoDrop One spectrophotometer (Thermo Fisher Scientific, Waltham, MA, USA). The RNA integrity numbers were measured with an Agilent Bioanalyzer with the Agilent RNA 6000 nano or pico kit (Agilent Technologies, Santa Clara, CA, USA) following the protocol established by the manufacturer.

### 2.4 RNA sequencing

For participant set 1, sequencing libraries were constructed employing the TruSeq Stranded Total RNA with Ribo-Zero Gold LT sample prep kit (Illumina, San Diego, CA, USA) following the protocol established by the manufacturer. After ribosomal RNA was removed, reverse transcription was performed to prepare the libraries. The libraries were pooled after quantification by bioanalyzer analysis and fluorometry with the Qubit dsDNA HS assay kit and a Qubit 2.0 fluorometer (Thermo Fisher Scientific, Waltham, MO, USA). Sequencing of paired-end fragments (75 bp × 2) was performed on a NextSeq 500 sequencing platform (Illumina; BGI, Beijing, China) to a depth of 13–35 (average: 21) million fragments.

For participant set 2, sequencing libraries were constructed with the TruSeq RNA Library Preparation Kit v2 (Illumina), and sequencing was performed using ṯhe HiSeq 4000 System (Illumina; BGI, Beijing, China).

Data for each sample were separated to generate FASTQ files. Next-generation sequencing data were cleaned with cutadapt (version 1.8.3) [21] and cmpfastq_pe.pl (http://compbio.brc.iop.kcl.ac.uk/software/cmpfastq_pe.php). After a step of quality control, the filtered short reads were mapped to the reference genome (hg38) with STAR (version 2.5.1b) [22]. Strand-specific or non-strand-specific transcripts per million were calculated for each sample with RSEM (version 1.3.3.) [23] and were then normalized using the trimmed mean of M value method. *P*-values were calculated using a likelihood ratio test. Gene functional analysis was performed using an ingenuity pathway analysis (Qiagen, Hilden, Germany).

### 2.5 q-PCR

cDNA synthesis and q-PCR were conducted following our previous study [20]. In brief, cDNA was synthesized with a PrimeScript RT reagent kit (Takara Bio, Kusatsu, Japan) with oligo (dT) primers and 1 μg of total RNA. It was then mixed with SYBR Premix Ex *Taq*II (Takara Bio, Kusatsu, Japan) and specific primers. Amplification was conducted for 50 cycles with a StepOnePlus real-time PCR system (Thermo Fisher Scientific, Waltham, MO, USA). Each cycle included amplification for 15 s at 95 °C and 1 min at 60 °C. The primers we used are presented in Supplementary Table 2. All measurements were conducted in duplicate. To quantify the levels of candidate gene expression and those of Beta-2-microglobulin (*B2M*; the internal control), a calibration curve was created with 10, 5, 2.5, 1.25, 0.625, and 0.3125 fM synthetic DNA, which included the target sequence (Integrated DNA Technologies, Coralville, IA, USA). Fold change was calculated by the ratio of each gene to the B2M expression level of the internal control, with the mean value of the responders as 1. The synthetic DNA sequences we used are shown in Supplementary Table 3.

### 2.6 Statistical analysis

The distribution of participant age, SIGH-D score before starting treatment, antidepressant dose, and mRNA expression data from q-PCR were investigated with Student’s *t*-tests, whereas the distribution of participant sex was investigated with Fisher’s exact test with EZR v1.54 (http://www.jichi.ac.jp/saitama-sct/SaitamaHP.files/statmedEN.html) [24]. Holm’s multi-testing correction was performed for the q-PCR results (Supplementary Table 4). The significance level was set to 0.05.

### 2.7 Data analysis

#### 2.7.1 Classifier design by discrete bayes decision rule

The discrete Bayes classifier handles discrete data. *x*_1_ and *x*_2_ are two markers and their range is exclusively divided into divisions.

Suppose that $x_{1}$ has two divisions and $x_{2}$ has three divisions. Moreover, the discretized data of a patient belong to the first division $x_{1\left(1\right)}$ in marker $x_{1}$ and the third division $x_{2\left(3\right)}$ in marker *x*_2_ That is, $x=\left(x_{1\left(1\right)\;},{x_{2}}_{\left(3\right)}\right)$. Then, $\text{P}\left(x_{1\left(1\right)\;}|\omega_{1}\right)$ and $\text{P}\left(x_{2\left(3\right)\;}|\omega_{1}\right)$ are defined as follows:

$$\text{P}\left(x_{1\left(1\right)\;}|\omega_{1}\right)=\frac{n_{1\left(1\right)}^{1}}{n_{1\left(1\right)}^{1}+n_{2\left(3\right)}^{1}}$$

and

$$\text{P}\left(x_{2\left(3\right)\;}|\omega_{1}\right)=\frac{n_{2\left(3\right)}^{1}}{n_{1\left(1\right)}^{1}+n_{2\left(3\right)}^{1}}$$

where $n_{1\left(1\right)}^{1}$ denotes the number of training samples for $\omega_{1}$ belonging to the division $x_{1\left(1\right)}$ and $n_{2\left(3\right)}^{1}$ denotes the number of the training samples for $\omega_{1}$ belonging to the division $x_{2\left(3\right)}$ Then, the class-conditional probability $P\left(x_{1\left(1\right)\;},x_{2\left(3\right)}|\omega_{1}\right)$ for fx1 is given by

$$P\left(x_{1\left(1\right)\;},x_{2\left(3\right)}|\omega_{1}\right)=\text{P}\left(x_{1\left(1\right)\;}|\omega_{1}\right)P\left(x_{2\left(3\right)\;}|\omega_{1}\right)$$

Using $n_{1\left(1\right)}^{2}$ and $n_{2\left(3\right)}^{2}$ for $\omega_{2}$, we similarly get

$$P\left(x_{1\left(1\right)\;}|\omega_{2}\right)=\frac{n_{1\left(1\right)}^{2}}{n_{1\left(1\right)}^{2}+n_{2\left(3\right)}^{2}}$$

and

$$P\left(x_{2\left(3\right)\;}|\omega_{2}\right)=\frac{n_{2\left(3\right)}^{2}}{n_{1\left(1\right)}^{2}+n_{2\left(3\right)}^{2}}$$

$P\left(x_{1\left(1\right)\;},x_{2\left(2\right)}|\omega_{2}\right)$ for $\omega_{2}$ is also given by

$$P\left(x_{1\left(1\right)\;},x_{2\left(3\right)}|\omega_{2}\right)=P\left(x_{1\left(1\right)\;}|\omega_{2}\right)P\left(x_{2\left(3\right)\;}|\omega_{2}\right)$$

The posterior probabilities of classes $\omega_{i}$ are given as follows:

$$P\left(\omega_{i}|x\right)=\frac{P\left(x_{1\left(1\right)\;},x_{2\left(3\right)}|\omega_{i}\right)}{P\left(x_{1\left(1\right)\;},x_{2\left(3\right)}|\omega_{1}\right)+P\left(x_{1\left(1\right)\;},x_{2\left(3\right)}|\omega_{2}\right)}$$

In this study, we assumed that in the *a priori* probability, $P\left(\omega_{i}\right),P\left(\omega_{1}\right)=P\left(\omega_{2}\right)$. The patient was then assigned to the class with the maximum posterior probability. For additional details, refer to previous studies [25,26].

#### 2.7.2 Marker selection

The following eight candidate genes were used to predict antidepressant response: *ISG15*, *RSAD2*, *HERC5*, *IFIT1*, *IFI6*, *IFI44*, *IFI44L*, and *IFIT3*. Among them, the combination of two markers was selected under conditions and examined. There were 30 training samples from participant set 1 and 34 test samples from participant sets 2 and 3. Note that marker selection was performed using only training samples.

We used the leave-one-out method [27] to identify an optimal combination of markers. According to the leave-one-out method, one training sample was selected as a sub-test sample from the 30 training samples and the remaining 29 training samples were assigned as sub-training samples (Supplementary Figure 1).

In pattern recognition fields, markers cannot be selected based on their individual effectiveness. Therefore, the combination of markers should be carefully selected. For this purpose, we first selected one combination of two markers. The discrete Bayes classifier was designed using 29 sub-training samples, and the re-substitution estimate for the candidate marker combination was obtained by reclassifying the 29 sub-training samples.

Next, feature criteria were calculated. Note that one sub-test sample was not used. This process was then repeated until all candidate marker combinations (_8_C_2_ = 28) had been evaluated. Among the 28 two-marker combinations, one marker combination with either maximal sensitivity subject to a specificity of ≥50% or a maximal F1 measure was selected to predict antidepressant response. Using the leave-one-out method, the above process was repeated 30 times (i.e., until each training sample had been selected once only as a sub-test sample). As a result, the most frequent combination of two markers identified in the leave-one-out loop was considered the optimal combination of markers. When the number of markers was three, marker selection was conducted again according to the same procedure.

## 3 Results

To identify genetic markers predictive of treatment response, we first conducted a genome-wide gene expression analysis without an *a priori* hypothesis. Among the untreated patients with MDD of participant set 1, we retrospectively assessed the treatment response after 6–8 weeks and divided them into the treatment responder (n = 15) and treatment non-responder groups (n = 15). We then performed RNA-Seq analysis using RNA obtained before starting treatment. Patient demographic data are shown in Table 1. There were no differences in sex, age, SIGH-D score before starting treatment, or antidepressant dose at the time of treatment response determination between groups. The SIGH-D score after treatment was significantly lower in the responder group than in the non-responder group (P = 3.22E-08).

**Fig. 1:**
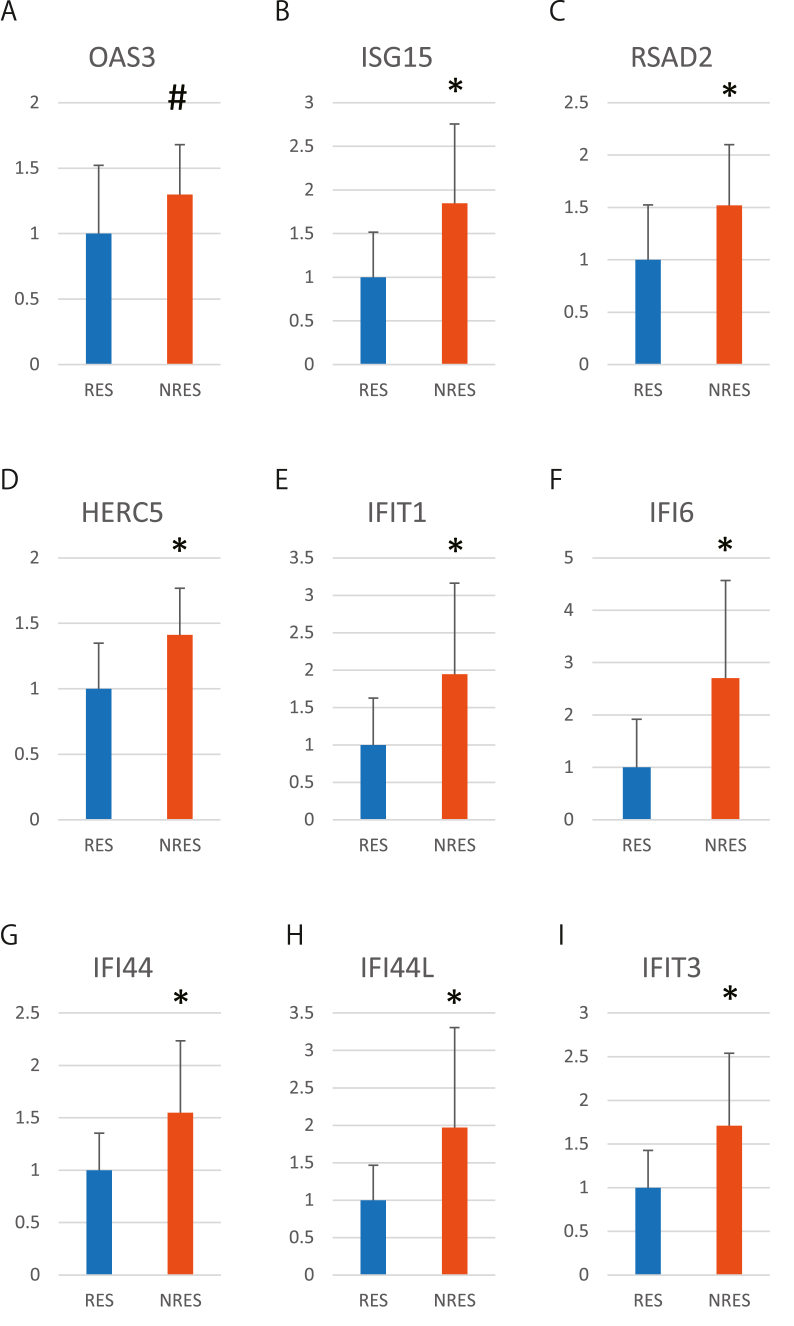
Comparison of candidate gene expression between responders and non-responders in participant set 1.1A-1I represent gene expression levels of OAS3, ISG15, RSAD2, HERC5, IFIT1, IFI6, IFI44, IFI44L, and IFIT3, respectively. Data are shown as mean ± standard deviation. Each vertical axis indicates a fold change. *P < 0.05 (unpaired Student’s t-test) and #P = 0.08 for RES versus NRES. Details of the p-values are shown in Supplementary Table 4. Abbreviations: RES, responder; NRES, non-responder; q-PCR, qualitative real-time polymerase chain reaction.

When we compared responders and non-responders, we identified 19 genes (P < 0.01 and a fold change of >1.5; Table 2). Among the 19 genes, the top 10 genes with an average count of 50 or more were validated using q-PCR. We found significant expression changes in eight genes (P < 0.05) (Fig. 1); however, *OAS3* did not show significant changes according to q-PCR (P = 0.08), and *SIGLEC1* was not successfully amplified using q-PCR. Thus, the remaining eight genes were selected as candidate predictive markers of antidepressant responsiveness.

**Table 2:** Genes with altered expression in non-responders of participant set 1 compared with those in responders based on RNA-Seq.

Gene	Gene name	Participant set 1	
		Log FC	*P*-value
*OAS3*	2'-5'-Oligoadenylate synthetase 3	0.864	0.0001
*ISG15*	ISG15 ubiquitin-like modifier	0.923	0.0001
*RSAD2*	Radical *S*-adenosyl methionine domain containing 2	0.905	0.0001
*HERC5*	HECT and RLD domain containing E3 ubiquitin protein ligase 5	0.795	0.0002
*IFIT1*	Interferon-induced protein with tetratricopeptide repeats 3	1.161	0.0002
*SIGLEC1*	Sialic acid-binding Ig-like lectin 1	1.178	0.0003
*IFI6*	Interferon-alpha inducible protein 6	0.908	0.0005
*GYPE*	Glycophorin E (MNS blood group)	−0.6	0.0007
*IFI44*	Interferon-induced protein 44	0.704	0.0012
*IFI44L*	Interferon-induced protein 44 like	0.997	0.0013
*IFIT3*	Interferon-induced protein with tetratricopeptide repeats 3	0.799	0.0018
*LRRC2*	Leucine-Rich repeat containing 2	0.707	0.0021
*EPHX1*	Epoxide hydrolase 1	0.631	0.0023
*RAP1GAP*	RAP1 GTPase activating protein	1.959	0.0023
*RPS26*	Ribosomal protein S26	1.012	0.0025
*OASL*	2'-5'-Oligoadenylate synthetase like	0.641	0.0025
*ACCS*	1-Aminocyclopropane-1-carboxylate synthase homolog (inactive)	−0.736	0.0034
*MGC70870*	*C*-terminal-binding protein 2 pseudogene	−0.731	0.005
*SLC14A1*	Solute carrier family 14 member 1 (Kidd blood group)	0.696	0.0071

Abbreviations: FC, fold change.

We then examined the untreated patients with MDD from an independent institution (participant set 2), whom we retrospectively divided into a treatment response group (n = 11) and treatment-resistance group (n = 5) after 8 weeks and performed comprehensive expression analysis without an *a priori* hypothesis using RNA-Seq data obtained before starting the treatment. The demographic data are presented in Table 1. There were no differences in sex, age, SIGH-D score before treatment initiation, or antidepressant dose at the time of treatment response determination after treatment between groups. The SIGH-D score after treatment was significantly lower in the responder group than in the non-responder group (P = 1.64E-05). The *P*-values and fold changes of eight candidate genes from participant set 1 are shown in Supplementary Table 5. We also identified 87 genes that met the criteria of *P*-values of <0.01 and fold changes of >1.5 in the responders compared with those in the non-responders according to the genome-wide gene expression analysis without an *a priori* hypothesis (Supplementary Table 6).

Furthermore, among the patients with MDD from another independent institute (participant set 3), we retrospectively examined the treatment response after 4–12 weeks and divided the patients into the responder (n = 11) and non-responder (n = 7) groups. The demographic data are provided in Table 1. There were no differences in sex, age, SIGH-D score before treatment, or antidepressant dose between groups. q-PCR was performed for these eight candidate genes identified from participant set 1 (Supplementary Figure 2).

Marker selection was then performed for eight candidate genes. In marker selection, feature criteria, such as accuracy, sensitivity, and F1 measure, were estimated using the discrete Bayes classifier designed with training samples from participant set 1. Varying the number of markers from two to three, all combinations of these eight genes were evaluated in the training samples. According to the feature criteria, two combinations were selected (Table 3). The two combinations were evaluated using this discrete Bayes classifier in the independent test samples from participant sets 2 and 3 (Table 3). The combination of *HERC5* and *IFI6* demonstrated high accuracy (79%). The combination of *HERC5*, *IFI6*, and *IFI44* exhibited the highest accuracy (sensitivity of 91%, specificity of 75%, and accuracy of 85%).

**Table 3:** Discrimination performance for identifying responders in training and test samples.

	Combination of markers	Accuracy	Sensitivity	Specificity	Precision	F1 measure
Discrimination performance for training samples (participant set 1) RES; n = 15, NRES; n = 15	*HERC5, IFI6*	0.83	0.93	0.73	0.78	0.85
	*HERC5, IFI6, IFI44*	0.90	0.93	0.87	0.88	0.90
Discrimination performance for test samples (participant sets 2 and 3) RES; n = 22, NRES; n = 12	*HERC5, IFI6*	0.79	0.91	0.58	0.80	0.85
	*HERC5, IFI6, IFI44*	0.85	0.91	0.75	0.87	0.89

Abbreviations: RES, responder; NRES, non-responder.

Finally, for functional analysis, we performed pathway analysis using 19 candidate genes identified in the comprehensive analysis of participant set 1. In the ingenuity canonical pathway, interferon signaling (P = 1.38E-08) and the role of hypercytokinemia/hyperchemokinemia in the pathogenesis of influenza (P = 4.79E-07) were significantly enhanced in non-responders relative to responders (Supplementary Table 7). Similar results were obtained using the 87 genes identified in participant set 2, an independent sample (Supplementary Table 8). These results suggested that markedly enhanced cytokine levels and interferon signaling were present in the antidepressant non-responder group.

In the analysis of diseases and biofunctions, the altered expression of genes associated with inflammatory and infectious diseases, including replication of hepatitis C (participant set 1; P = 5.81E-07, participant set 2; P = 4.24E-07) and coronavirus disease (COVID-19) (participant set 1; P = 2.7E-11, participant set 2; P = 3.95E-10), was observed (Supplementary Tables 9 and 10).

## 4 Discussion

Immune responses and inflammation have been implicated in the pathogenesis of MDD. We performed a comprehensive gene expression analysis without an *a priori* hypothesis using blood samples from patients with MDD who were not yet taking antidepressants associated with treatment response. The expression levels of the identified genes were validated using q-PCR. Gene expression was further validated using independent samples by RNA sequencing (participant set 2) or q-PCR (participant set 3). Although we could not compare gene expression levels directly among samples from different facilities owing to different protocols for RNA purification and analysis, we could separate responders from non-responders with a discrimination accuracy of 85% using a combination of three genes, *HERC5*, *IFI6,* and *IFI44*, for independent test samples. Most importantly, we could classify the treatment response in completely independent test samples obtained from different facilities using genes identified in training samples via the data-driven approach. Our results suggested that the combination of these genes could be used as a predictive marker of treatment response. In recent depression treatment guidelines, antidepressant medication is only one option for first-line treatment, as is psychological therapy [28,29]. Prediction of potential antidepressant non-responders can be useful for a shared decision-making process. During the treatment selection phase, psychological therapy alone or in combination of psychological treatment and pharmacotherapy may be chosen, or electroconvulsive therapy may be recommended for cases of more severe depression.

We performed pathway analysis using identified gene sets and found that gene expression related to hypercytokinemia and interferon signaling was increased in non-responders. These results are in line with those of previous reports that inflammatory signals are involved in antidepressant responsiveness [8–11].

The gene clusters associated with hypercytokinemia and interferon signaling were significantly increased in treatment non-responders in three independent sample groups, and this provides a basis for speculating on the pathological mechanisms underlying antidepressant responsiveness. Interferon signaling induces depression and may be associated with increases in cytokine levels. For example, in patients with hepatitis C who were depressed after receiving interferon-alpha, increased spinal fluid IL-6 and monocyte chemotactic protein 1 levels were noted. Spinal fluid IL-6 was also negatively correlated with 5-hydroxyindole acetic acid, a serotonin metabolite [30]. Interferon-alpha has also been reported to cause reduced serum brain-derived neurotrophic factor levels, which may contribute to the mechanism underlying depression [31]. Peripheral interferon increased the expression of interferon-stimulated genes such as *Rsad2*, *Isg15,* and *Ifit3* in the cortical microglia of mice and induced microglial activation [32]. In humans, microglia are considered to play crucial roles in various psychiatric disorders, including depression [33].

Regarding inflammatory cytokines and treatment responsiveness, as mentioned in the Introduction, several studies have demonstrated increased inflammatory cytokine levels in treatment-resistant depression. Therefore, the administration of anti-inflammatory agents has been proposed as a potential novel treatment for MDD. For example, depressive-like behavior and impaired neurogenesis in the hippocampus induced by interferon-alpha can be improved by minocycline administration in mice [34]. In humans, a large meta-analysis of randomized controlled trials (RCTs) of anti-inflammatory treatments reported the antidepressant effects of non-steroidal anti-inflammatory drugs (NSAIDs) and anti-inflammatory drugs [[35], [36], [37]]. Moreover, the tolerability of NSAIDs was reported to be superior to that of a placebo [37]. In contrast, large RCTs have yielded negative results on the preventive effect of small doses of aspirin on depression [38]. Thus, anti-inflammatory drugs are considered to have promising antidepressant effect; however, the selection of target patients is also important. For example, if the gene expression changes identified in this study are established as biomarkers and anti-inflammatory drugs are introduced after the identification of patients who are likely to be resistant to treatment, more effective therapeutic interventions are possible.

Notably, COVID-19 was identified in the analysis of diseases and biofunctions. It has been reported that 23.0% of COVID-19 patients had pre-existing depression [39]. Patients with psychiatric disorders, including depression, have increased mortality, hospitalization, and intensive care unit admission after severe acute respiratory coronavirus 2 infection [40]. It has also been reported that the levels of inflammatory markers, including IL-6, are increased in patients with COVID-19 [41]. The involvement of immune dysfunction in the development of depression has also been proposed [42]. Recently, *OAS3*, a gene identified in this study, was noted to be associated withCOVID-19 severity according to a GWAS analysis [43]. COVID-19-induced depression may be a factor affecting antidepressant resistance. In contrast, SSRIs may prevent the development of COVID-19 [44]. It will be important to monitor whether COVID-19 patients with depression respond well to antidepressants.

This study has several limitations. Firstly, the number of cases was small. However, depression is not a homogeneous disease but a heterogeneous syndrome. We previously reported that DNA methylation and gene expression profiles differ according to age of onset and sex [[45], [46], [47], [48]]. Rather than merely increasing the number of participants, it is also important to collect more distinctive and uniform cases in future studies. In this study, reproducible changes in gene expression were identified in patients from three independent institutes focusing on antidepressant responsiveness. The second limitation was that this was an observational study rather than a double-blind interventional study with a placebo; hence, the effects of a placebo and nocebo on treatment response cannot be ignored. Thirdly, it was difficult to standardize the classes and dosages of antidepressants and match the timing of assessment because this study was conducted in a real-world clinical practice setting to reduce the burden on patients. However, the fact that we were able to identify reproducible treatment response markers among patients may be a strength of this study. To address these limitations, multicenter, double-blinded, large-scale interventional studies need to be conducted. The fourth limitation is that the methods of RNA purification and expression analysis differed among institutions. In our previous study, when RNA-Seq was conducted with RNA purified using different methods, significant differences in housekeeping gene expressions were identified [20]. It is necessary to standardize RNA purification methods and establish discrimination criteria at each institution for clinical application as predictive markers of treatment responses. The final limitation is that only RNA analysis was performed. A more robust biomarker could be identified by setting up a complex biomarker with plasma or DNA methylation.

Despite these limitations, the results of this study form the basis for the development of predictive markers of the therapeutic response to depression and may provide insights into the development of novel therapeutic strategies for depression, such as anti-inflammatory drugs.

## Author contributions

Conceptualization: HY, SU, YO, TO, SN. Data curation: HY, RT, TK, KH, NY, CC, HO, YH, GO, MF, SN, MK, RH, YO, TO, SN. Formal analysis: HY, RT, CC, HO, YH. Funding acquisition: HY, TAK, SU, YO, TO, SN. Investigation: HY, RT, AK, SN, MK. Methodology: HY, RT, AK. Project administration: HY, SU, YO, TO, SN. Resources: HY, TK, AK, TS, MK, KH, NY, GO, MF, SN, MK, YO, TO, SN. Supervision: SU, YH, JI, SN, TAK, HN, SU, YO, TO, SN. Validation: HY, RT, AK, HO, YH. Visualization: HY, RT, HO, YH. Writing – original draft preparation: HY. Writing – review & editing: HY, RT, TK, AK, TS, MK, KH, NY, CC, SU, HO, YH, GO, MF, JI, SN, MK, TAK, RH, HN, SU, YO, TO, SN.

## Funding

This study was supported by the 10.13039/100009619Japan Agency for Medical Research and Development (AMED) (JP20dk0307076h0003 to SU, YO, TO, and SN and JP20dk0307075 to TAK). HY was partially supported by the 10.13039/501100001691Japan Society for the Promotion of Science (JSPS) KAKENHI program (20K07946); SENSHIN Medical Research Foundation; and grants from the Finding-Out & Crystallization of Subliminals (FOCS) project by the Yamaguchi University of Medicine.

## Declaration of competing interest

HY received honoraria and/or research grant support from Pfizer, Eisai, MSD, Sumitomo Dainippon Pharma, Mochida Pharmaceutical, Mylan, Viatris, Otsuka Pharmaceutical, and Yoshitomiyakuhin. GO received lecture fees from Otsuka Pharmaceutical, Pfizer Japan, and Sumitomo Dainippon Pharma. JI received speaker’s honoraria from Otsuka Pharmaceutical, Meiji Seika Pharma, Sumitomo Dainippon Pharma, Kyowa Pharmaceutical Industry, Shionogi, Mochida Pharmaceutical, Eisai, Mylan, Sawai Pharmaceutical, Novartis Pharma, Eli Lilly, MSD, Ono Pharmaceutical, Takeda Pharmaceutical, Janssen Pharmaceutical, Sanofi, Viatris, and Yoshitomiyakuhin. SN received honoraria and/or research grant support from Otsuka Pharmaceutical, Sumitomo Dainippon Pharma, Mochida Pharmaceutical, and Eisai. TAK received honoraria and/or research grant support from Otsuka Pharmaceutical, Mitsubishi Tanabe Pharma, Mochida Pharmaceutical, and Pfizer. TO received research support or speakers’ honoraria from, or has served as a consultant to Sumitomo Dainippon Pharma, Otsuka Pharmaceutical, Eisai, Pfizer, Eli Lilly, Janssen, Meiji Seika Pharma, Shionogi, Mitsubishi Tanabe Pharma, Takeda Pharmaceutical and Yoshitomiyakuhin. SN received honoraria and/or research grant support from Otsuka Pharmaceutical, Meiji Seika Pharma, Sumitomo Dainippon Pharma, Kyowa Pharmaceutical Industry, Shionogi, Mitsubishi Tanabe Pharma, Mochida Pharmaceutical, Eisai, Tsumura, Eli Lilly, MSD, Astellas, and Pfizer. The remaining authors declare no actual or potential conflicts of interest.
