# Supplementary figures and images for: Interferon signaling and hypercytokinemia-related gene expression in the blood of antidepressant non-responders
Source: Heliyon. 2023 Jan 16;9(1):e13059. doi: 10.1016/j.heliyon.2023.e13059 (PMC9876967; doi:10.1016/j.heliyon.2023.e13059)

Supplementary Figure 1.

A summary of statistical strategy.

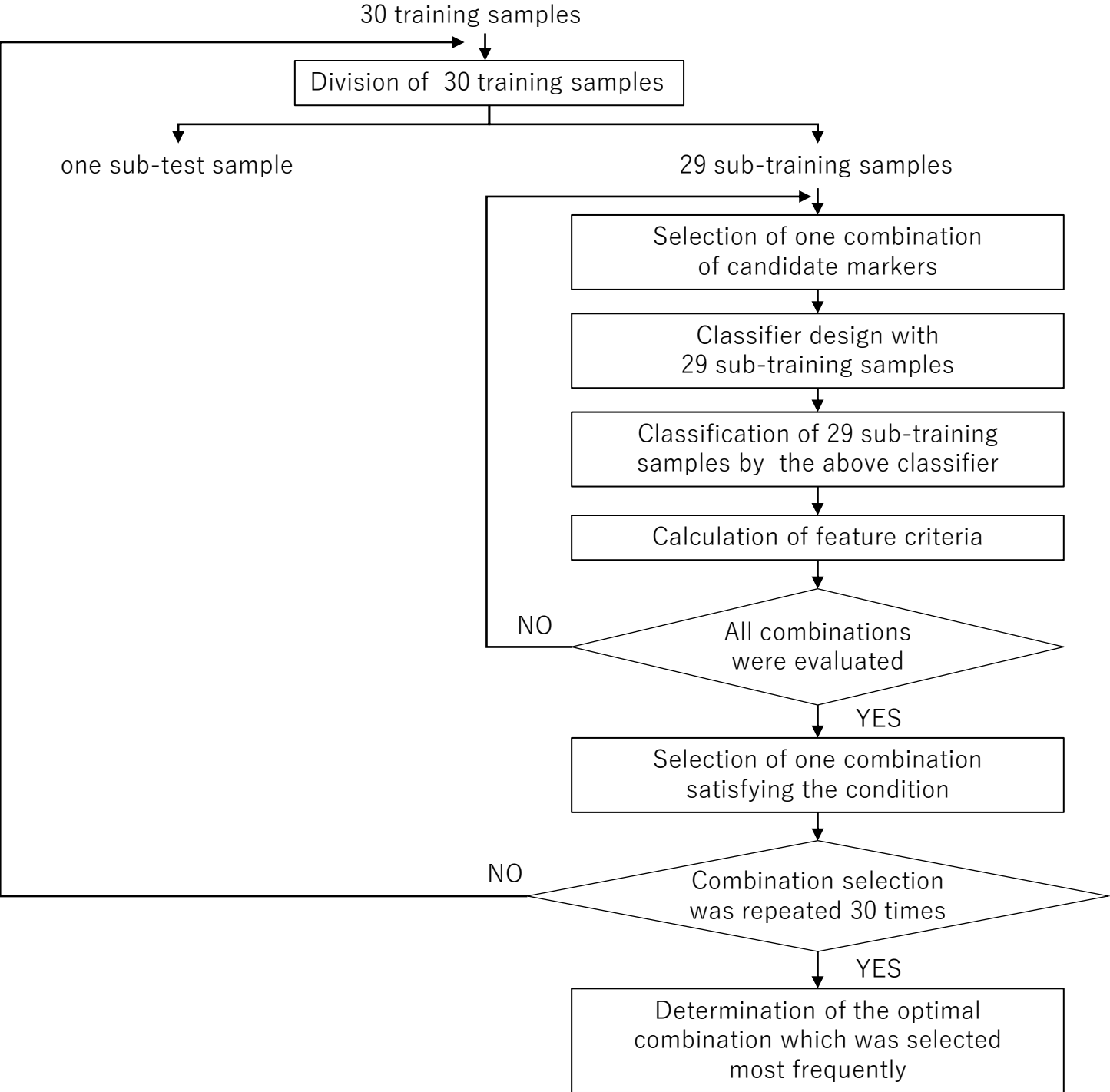

Supplement: Multimedia component 1 [file mmc1.pdf]
