# Supplementary material for: Interferon signaling and hypercytokinemia-related gene expression in the blood of antidepressant non-responders
Source: Heliyon. 2023 Jan 16;9(1):e13059. doi: 10.1016/j.heliyon.2023.e13059 (PMC9876967; doi:10.1016/j.heliyon.2023.e13059)
Supplement: Multimedia component 2 [file mmc2.pdf]

Supplementary Figure 2

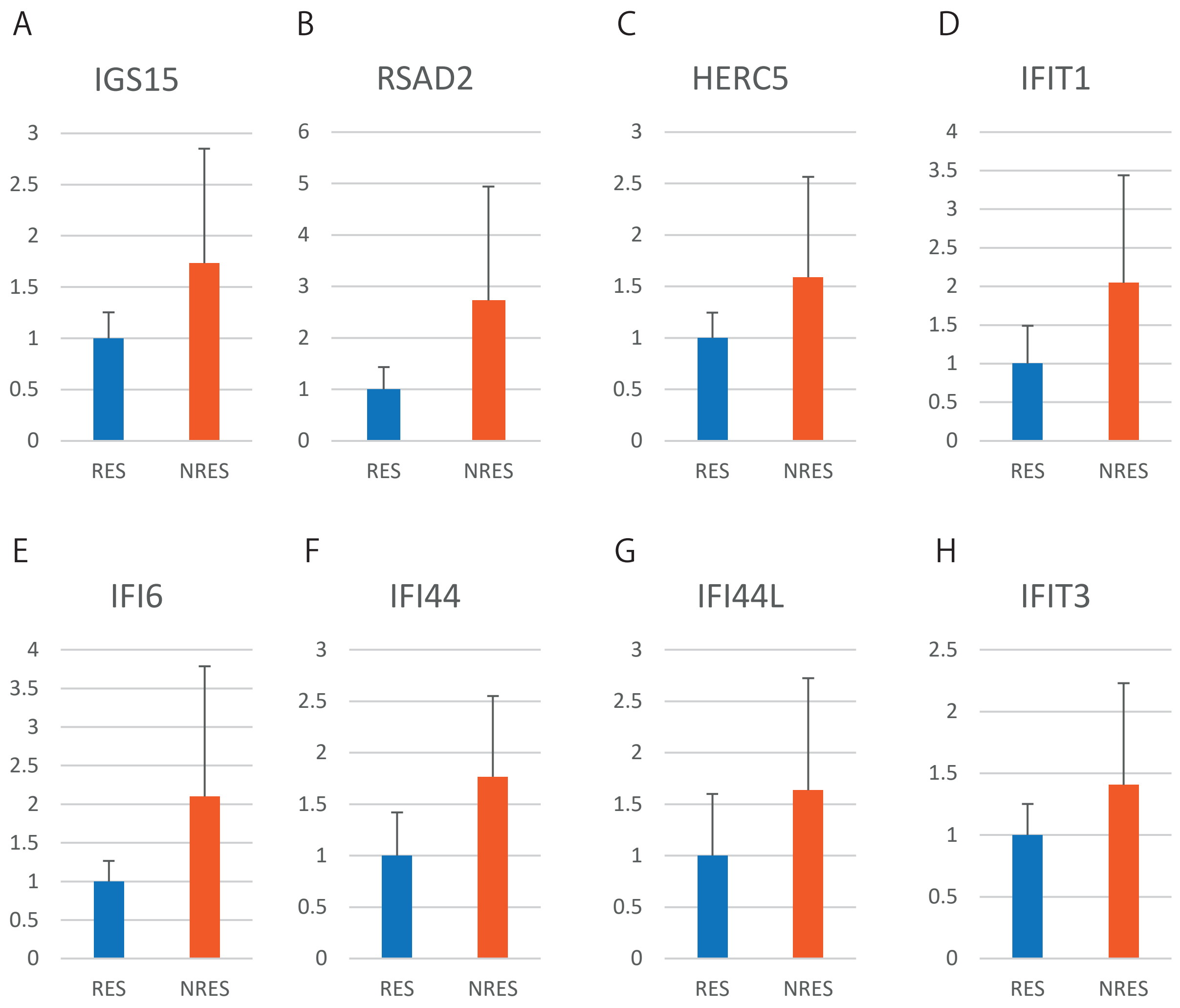

Comparison of candidate gene expression between responders and non responders in participant set 3

2A-2H represent gene expression levels of ISG15, RSAD2, HERC5, IFIT1, IFI6, IFI44, IFI44L, and IFIT3, respectively.

Data are shown as the mean ± standard deviation.

Abbreviations: RES, responder; NRES, non responder
